# When It Is Not Measured, How Then Will It Be Planned for? WaSH a Critical Indicator for Universal Health Coverage in Kenya

**DOI:** 10.3390/ijerph17165746

**Published:** 2020-08-08

**Authors:** Thelma Zulfawu Abu, Susan J. Elliott

**Affiliations:** Department of Geography and Environmental Management, University of Waterloo, 200 University Avenue West, Waterloo, ON N2L 3GI, Canada; susan.elliott@uwaterloo.ca

**Keywords:** WaSH, universal health coverage, quality care, healthcare facility, LMICs

## Abstract

The quality and safety of healthcare facility (HCF) services are critical to achieving universal health coverage (UHC) and yet the WHO/UNICEF joint monitoring program for water supply, sanitation and hygiene report indicates that only 51% and 23% of HCF in Sub-Saharan Africa have basic access to water and sanitation, respectively. Global commitments on improving access to water, sanitation, hygiene, waste management and environmental cleaning (WaSH) in HCF as part of implementing UHC have surged since 2015. Guided by political ecology of health theory, we explored the country level commitment to ensuring access to WaSH in HCFs as part of piloting UHC in Kisumu, Kenya. Through content analysis, 17 relevant policy documents were systematically reviewed using NVIVO. None of the national documents mentioned all the component of WaSH in healthcare facilities. Furthermore, these WaSH components are not measured as part of the universal health coverage pilot. Comprehensively incorporating WaSH measurement and monitoring in HCFs in the context of UHC policies creates a foundation for achieving SDG 6.

## 1. Introduction

Accessing quality health services is a challenge, especially in the global south. Lack of access to water, sanitation, hygiene, waste management and environment cleaning (WaSH) undermine the quality of services provided in healthcare facilities [1,2]. The absence or inadequacy of safe WaSH in healthcare facilities compromises infection prevention and control, patient safety and child and maternal health [3]. Meanwhile, the WHO/UNICEF joint monitoring program for water supply, sanitation and hygiene reported that in Sub-Saharan Africa (SSA), only 51 percent of healthcare facilities have access to basic water services and 23 percent have access to basic sanitation services. Forty-one percent of healthcare facilities have basic waste management services. Data on hygiene and environmental cleaning in healthcare facilities were inconclusive due to inadequate monitoring [1]. Similarly, Cronk and Bartram [2] evaluated the environmental conditions of healthcare facilities in 78 low- and middle-income countries (LMICs) and found that only two percent of the healthcare facilities provided water, sanitation, hygiene and waste management services. Also, ensuring access to WaSH in healthcare facilities extends beyond disease control to issues of dignity and respect. For example, women after childbirth in healthcare facilities require a clean bathroom with running water to maintain their personal hygiene. Kohler, Renggli, & Lüthi [4] in a comparative study in India and Uganda sought to address the gender gap in access to WaSH in healthcare facilities. They undertook a needs assessment in hygiene and sanitation issues during menstruation and childbirth among women in selected maternal ward and inpatient facilities which were run by government. WaSH in healthcare facilities were assessed based on hygiene and health, security and safety, privacy, accessibility, comfort and menstrual hygiene management. From their study, lack of safe WaSH infrastructure and menstrual hygiene facilities were a burden for women in both countries. In addition, Gon et al. in 2016 engaged in a study to investigate the status of water and sanitation in relation to childbirth in healthcare facilities and homes. From their study, less than 50 percent of all delivery facilities and homes had access to WaSH in all countries [5]. For example, in Kenya, 18 percent of women delivered with improved access to water and sanitation. Furthermore, climate change and variability and conflicts burden the functioning of WaSH in healthcare facilities. First, 90 percent of disasters in SSA, especially the horn of Africa, are water-related [6]. Prolonged drought and floods have affected the quantity and quality of water available [7,8]. Second, displaced people face WaSH related challenges and these events increase health risks and disease outbreaks such as cholera [9,10].

Prior global commitments on ensuring access to WaSH were concentrated at the household level to the neglect of institutions. The widespread effects of Ebola in 2014 even in healthcare facilities leading to the loss of several healthcare workers [11,12,13] and the subsequent World Health Organization assessment on WaSH in healthcare facilities in 2015 initiated discussions and led to several global commitments to address this challenge of infection prevention and control in healthcare facilities. At the global stage currently, significant efforts towards ensuring access to WaSH have included and prioritized public spaces such as healthcare facilities. This is included in the sustainable development goals (SDG). Goal 6 seeks to ensure access to water and sanitation. Targets 6.1 and 6.2 of the SDGs highlight the need to expand WaSH monitoring by relevant stakeholders in non-household settings, such as healthcare facilities. Similarly, Goal 3 seeks to ensure healthy lives and promote wellbeing for all at all ages. Target 3.8 highlights achieving universal health coverage which does not just incorporate reducing the financial burden of people, but further ensuring quality essential healthcare services for all. Similarly, in 2015, world leaders adopted the Sendai framework for disaster risk reduction (DRR) and one of its targets is to substantially reduce disaster damage to critical infrastructure and disruption of basic services, among them health facilities through developing their resilience by 2030 [14]. This framework was a paradigm shift from managing disasters to disaster risk reduction. Achieving this target means ensuring the effectiveness and efficiency of all the components of a health system, including WaSH.

In March 2018, as part of the launch of International Decade for Action “Water for Sustainable Development 2018–2028”, the UN Secretary General also made a global call to action for WaSH in all healthcare facilities [15]. In response, various ministers of state signed the World Health Assembly resolution on WaSH in healthcare facilities as part of the implementation of universal health coverage scheme. In addition, various assessment tools, healthcare facility guidelines and frameworks on WaSH were published by the global community especially World Health Organization. However, it is evident from research that socially and institutionally driven challenges such as lack of data and knowledge are major hindrances to improved service provision such as healthcare in SSA [10,16,17]. For instance, Adjei, Sambu & Smiley [18] explored historical and emerging policies and institutional arrangements surrounding urban water supply in Sub-Saharan Africa. The persistent lack of water in urban areas was attributed to weak institutional arrangements and poor enforcement of legislations. The authors recommended the need for institutional rectification to achieve the sustainable development goals by 2030. Similarly, Maina et al. [3] in their study on the role of WaSH on antimicrobial resistance in healthcare facilities in Kenya highlighted the need for government institutional support for healthcare managers to enable them achieve access to basic WaSH in healthcare facilities. It is evident from research that the availability and enforcement of regulations such as policies and legislations on an agenda enhance their achievement [19]. Guo & Bartram [20] in their investigation on the predictors for water quality in rural healthcare facilities concluded that the presence of a protocol for operation and management in a facility was associated with safe water use. Following this, there is little research to understand the implementation process or the institutional arrangements of WaSH in healthcare facilities and the influence of global commitments on country level policy environment on ensuring access to WaSH in health facilities in SSA. Therefore, this paper reviews the framing of WaSH in healthcare facilities in relevant global and country—level institutional documents (policies, legislations, guides, plans and monitoring tools) using Kenya as a case study. Following the introduction, the second section explores the theoretical framing of this paper, the political ecology of health theory. The third section explores the study context, Kenya. The fourth section indicates the methods of data collection and analyses. The presentation of the results and discussion make up the fifth and sixth sections, respectively. The seventh section concludes the paper with a summary of the key points and emphasizing the relevance of WaSH in healthcare facilities to SDG 3 and SDG 6.

### 1.1. Theoretical Framework

Social theories provide a more comprehensive connection between determinants and processes of health and wellbeing [21,22,23]. The paper is guided by political ecology of health theory, which explores how power, politics, structures, agendas and/or agents shape the environment and health risks of populations [24,25]. This theory further explores how growing discourse on health at the global scale influence and shape local contexts such as policies development and implementation. The prioritization, implementation and management of WaSH interventions are political and power-laden at the global, national and local levels [26]. This theory has been useful in the study of prioritization and implementation of development projects and health and wellbeing of local populations [27,28,29]. It has also guided studies in healthcare services in LMICs [24] and privatization of water and its impacts on health and wellbeing [30].

### 1.2. Study Context

Kenya is an East African country with an estimated population of about 48 million [31]. The country has 47 counties. According to the Kenyan health policy 2012–2030, Kenya has an agenda to implement universal health coverage and achieve countrywide coverage by 2022. In 2018, the universal health coverage scheme was launched and currently piloted in four counties, Kisumu, Isiolo, Machakos and Nyeri. A policy brief written by Wangia & Kandie [32] and published by the ministry of health with a focus on quality of care and essential elements in attaining universal health coverage in Kenya indicated the need for appropriate water and sanitation infrastructure in healthcare facilities. According to the WHO/UNICEF joint monitoring program for water supply, sanitation and hygiene report based on 2016 data, only 65% of healthcare facilities in Kenya had access to basic water services. This served a population of 31, 784, 828 people. Healthcare facilities with limited and no water services were 17.6 percent and 16.8 percent, respectively. Concerning sanitation in healthcare facilities, monitoring and data collection was inadequate. Eighty-six percent of healthcare facilities had insufficient data and 14 percent of healthcare facilities recorded no sanitation services. Regarding hygiene, insufficient data for 99.6 percent of healthcare facilities was recorded. In addition, 0.4 percent of the healthcare facilities recorded no hygiene services. Only 33.1 percent of healthcare facilities recorded basic waste management services, 62.1 percent recorded limited services and 4.8 percent reported no waste management services. For environmental cleaning in healthcare facilities data were insufficient for comprehensive and conclusive analysis. From these data it is evident that access, regular monitoring and evaluation of WaSH in healthcare facilities are major challenges. Other researchers such as Bennett, Otieno, Ayers, & Odhiambo [33], Essendi et al. [34] and Maina et al. [3] have reported lack of WaSH in healthcare facilities in Kenya in their studies. In addition, at the community level, residents questioned the quality of healthcare delivery in hospitals without the appropriate WaSH infrastructure [35,36]. According to Wangia & Kandie [32], quality care is not yet a legal requirement and issues such as poor enforcement of legislation and minimal information on quality of care especially in private facilities will negatively impact achieving universal health coverage.

Other key challenge to accessing WaSH in healthcare facilities are climate variability and civil disruptions. The amount of rainfall affects the quantity and quality of water available for use in most marginalized communities. The struggle to access safe water is worsened in the face of climate variability. Floods from torrential rains and effects of drought from prolonged dry seasons have displaced many citizens, especially in rural and marginalized areas. As of September 2017, about 5.6 million Kenyan citizens were in need due to several episodes of drought [9]. Kenya has also recorded an increasing influx of migrants from neighboring countries greatly affected by drought. These people are further exposed to health hazards subsequently increase attendance at healthcare facilities. Kenya has a partial plan to support ensuring access to WaSH in health care facilities [10]. Despite progress and new initiatives, more needs to be done to understand and solve the challenge of lack of WaSH in healthcare facilities.

## 2. Methods

Qualitative content analysis was used to analyze the framing of WaSH in healthcare facilities in relevant documents for this paper. Relevant WaSH in healthcare facility documents such as policies, legislations, guidelines, plans and monitoring tools were gathered for this research from May 2019 to June 2020. Documents included in this research were accessed using two methods. First, desktop searches were conducted to identify and access current and operational WaSH in healthcare facility documents. Desktop searches on key phrases like “WaSH in healthcare facilities”, “quality care” and “universal health coverage” were done using google and google scholar. The websites of the Ministry of Health, Kenya, World Health Organization, WHO/UNICEF joint monitoring program for water supply, sanitation and hygiene as well as the official website for WaSH in healthcare facilities were searched for relevant documents. Second, the Ministry of Health, Kisumu County office, Kenya was contacted in person by researchers from June 2019–September 2019 for relevant documents on WaSH in healthcare facilities. Current operational documents guiding the implementation and monitoring of WaSH in healthcare facilities, quality healthcare and the piloted universal health coverage as of September 2019 were sought at the ministry. Documents included in this study were based on three criteria after been carefully screened. First documents comprehensively indicated WaSH in healthcare facilities or/and health care (quality care and universal health coverage) as their focus. Second, current and operational national documents with an agenda on WaSH in healthcare facilities, quality care in healthcare facilities and universal health coverage were also considered. Third, document was listed by relevant key stakeholders identified and interviewed at the ministry of health, Kisumu county office. The documents included in this study were published from 2007 to 2019. Documents prior to 2015 when the upsurge in campaigns for WaSH in HCFs and UHC were included because they set the foundation for drafting current WaSH in HCF guidelines and policies. Table 1 shows a list of relevant documents included in this research. First, the documents were categorized based on scale—global and national. Second, based on the purpose of the document—legislation, policy, guidelines, monitoring tool and plans. In total, 17 documents were included, five (5) global level documents and eight (12) national level documents regulating issues of WaSH in healthcare facilities. Two of the twelve national documents are county level documents. Kenya has a decentralized government system and the counties have the power to contextualize national policies or develop policies that meet their needs.

### Coding Frame

A coding frame (Table 2) was developed to guide the coding process. The frame was guided by the logic framework (input, activities, output and impact), heuristic framework (agenda setting, formulation, implementation and evaluation) [37] and policy triangle (grounded in a political economy perspective and considers actors, context, process and content shape policymaking) [38]. The authors adapted the WaSHFIT conceptual framework [39,40]. It is a framework designed to help implementers identify risks in healthcare facilities and it provides practical tools and templates for managing WaSH and facilities. Themes developed for coding were first guided by the water–health nexus. Cook & Bakker [41] define water security as “sustainable access on a watershed basis to adequate quantities of water, of acceptable quality, to ensure human and ecosystem health”. This definition embodies two SDGs, SDG 3—good health and wellbeing, of particular interest to this research is target 3.8 (Achieve universal health coverage, including financial risk protection, access to quality essential healthcare services and access to safe, effective, quality and affordable essential medicines and vaccines for all) and SDG 6, clean water and sanitation for all. In addition, the key components of WaSH—water, sanitation, hygiene, waste management and environmental cleaning were adapted from the WHO/UNICEF joint monitoring program for water supply, sanitation and hygiene. Key indicators for monitoring WaSH in healthcare facilities and categorized as improved, basic, limited and no service [1]. Guided by this coding frame, a coding schedule (Table 3, Table 4 and Table 5) was developed for coding. Content analysis was done deductively using NVIVO 12. Key phrases like WaSH in healthcare facilities, universal health coverage, WaSH in healthcare facility stakeholders and quality care were coded.

## 3. Results

This research explored the framing of WaSH in healthcare facilities in relevant global and national policies, guidelines, monitoring tools and legislations. From the content analysis, five (5) global documents comprehensively mentioned WaSH in healthcare facilities. Two national level documents mentioned water, sanitation and hygiene in phrases or sentences while environmental cleaning and waste management were excluded.

“*The core indicators define “basic” service levels for water, sanitation, hygiene, health care waste management and environmental cleaning in health care facilities*”

(Core questions and indicators for monitoring WASH in health care facilities in the Sustainable Development Goals)

The need to ensuring access to water, sanitation and hygiene in health care facilities was mentioned:

“*Ensure that all new health facilities are appropriately designed and constructed with reliable water supply and environmental sanitation and hygiene facilities, including toilet and hand-washing facilities, taking into account gender, age and disability considerations*”

(Kenya Environmental Sanitation and Hygiene policy 2016–2030).

“*Facility design and planning should ensure the following: Adequate supply of safe water, Adequate floor space for beds, Adequate space between beds, Adequate hand-washing facilities, Adequate sanitary facilities*”

(National Infection Prevention and Control Guidelines for Health Care Services in Kenya, 2010).

### 3.1. Global WaSH in Healthcare Facility Documents Serve as Guides for National Implementation

The global documents serve as a guide for national WaSH in healthcare facility implementation. They also specify the core areas of WaSH in healthcare facilities that need facility managers and implementers attention:

“*to develop and implement a road map according to national context so that every healthcare facility in every setting has, commensurate with its needs: safely managed and reliable water supplies; sufficient, safely managed and accessible toilets or latrines for patients, caregivers and staff of all sexes, ages and abilities; appropriate core components of infection prevention and control programmes, including good hand hygiene infrastructure and practices; routine, effective cleaning; safe waste management systems, including those for excreta and medical waste disposal; and, whenever possible, sustainable and clean energy*”

(A72_R7 WaSH in Healthcare Facilities Resolutions).

The global WaSH in healthcare facilities documents also set a monitoring standard for countries given in-country monitoring indicators on WaSH in healthcare facilities are often not comprehensive:

“*In support of SDG monitoring and to allow for comparable data to be generated within and between countries, a core set of harmonized indicators and questions that address basic WASH services in health care facilities that will be applicable in all contexts is needed*”

(Core Questions for monitoring WaSH in healthcare facilities in the Sustainable Development Goals).

The individual components of WaSH were highlighted in the documents assessed. The various components are outlined below.

#### 3.1.1. Water

Recommended water sources for healthcare facilities include piped water, boreholes or tube wells, protected dug wells, protected springs, rainwater and packaged or delivered water. The theme water in healthcare facilities was mentioned in nine (9) documents of which four were national documents. Some documents highlighted the need for water in healthcare facilities:

“*Sufficient water-collection points and water-use facilities are available in the health center to allow convenient access to, and use of, water for drinking, food preparation, personal hygiene, medical activities, laundry and cleaning*”

(Essential Environmental Health Standards in Healthcare).

The types of water systems in healthcare facilities were also mentioned in some documents:

“*Improved water sources in healthcare settings include piped water, boreholes/tube wells, protected wells, protected springs, rainwater and packaged or delivered water*”

(WaSHFIT, A practical guide for improving quality of care through WaSH in HCFs).

At the national level, the Water Act mentions the provision of water in healthcare facilities:

“*Nothing in this section prohibits—(a) the provision of water services by a person to his employees; or (b) the provision of water services on the premises of any hospital, factory, school, hotel, brewery, research station or institution to the occupants thereof, in cases where the source of supply of the water is lawfully under its control or where the water is supplied to it in bulk by a licensee*”

(Water Act Cap 372).

#### 3.1.2. Sanitation

Recommended sanitation infrastructure includes flush/pour flush to piped sewer system, septic tanks or pit latrines; ventilated improved pit latrines, composting toilets or pit latrines with slabs. Sanitation in healthcare facilities was highlighted in five (5) global documents and three (3) national documents. Basic sanitation service was defined as follows:

“*Basic sanitation services Definition: Proportion of health care facilities with improved and usable sanitation facilities, with at least one toilet dedicated for staff, at least one sex-separated toilet with menstrual hygiene facilities, and at least one toilet accessible for users with limited mobility*”

(Core questions in monitoring WaSH in healthcare facilities in the Sustainable Development Goals).

The maintenance of sanitary infrastructure was highlighted.

“*ensuring houses, institutions, hospitals and other public places maintain environment to the highest level of sanitation attainable to prevent, reduce or eliminate environmental health risks*”

(Kenya Health Act No.21 of 2017).

#### 3.1.3. Hygiene

Hygiene infrastructure include sink with tap, water tank with tap, bucket with tap or similar device, alcohol based hand rub dispensers. Hygiene in healthcare facilities was highlighted in eight documents analyzed. Three (3) national level documents and five (5) global documents. Hygiene was defined as:

“*Basic hygiene services Definition: Proportion of health care facilities with functional hand hygiene facilities available at one or more points of care and within 5 meters of toilets*”

(Core questions for monitoring WaSH in healthcare facilities in the Sustainable Development Goals).

The importance of hygiene facilities was also highlighted in some documents, for example:

“*Hand hygiene is the single most important IPC precaution and one of the most effective means to prevent transmission of pathogens associated with health care services. Appropriate hand hygiene must be carried out upon arriving at and before leaving the health care facility, as well as in the following circumstances*”

(National infection Prevention and Control Guidelines for Health Care Services in Kenya)

#### 3.1.4. Waste Management

Waste management in healthcare facilities was highlighted in nine (9) documents. Different types of waste are generated from various sectors of the healthcare facility as a result waste segregation was highly illustrated in the documents:

“*The four major categories of health-care waste recommended for organizing segregation and separate storage, collection and disposal are*:

- sharps (needles, scalpels, etc.), which may be infectious or not
- non-sharps infectious waste (anatomical waste, pathological waste, dressings, used syringes, used single-use gloves)
- non-sharps non-infectious waste (paper, packaging, etc.)
- *hazardous waste (expired drugs, laboratory reagents, radioactive waste, insecticides, etc.)*”

(Essential Environmental Health Standards in Healthcare).

It is recommended colors and images be used to identify waste containers and waste should be appropriately disposed by incineration, autoclaving and burial in a lined, protected pit. The repercussions of improper healthcare waste management were mention.

“*Review medical waste management guidelines for health care facilities to protect public health and safety, provide a safer working environment, minimize waste generation and environmental impacts of medical waste disposal and ensure compliance with legislative and regulatory requirements*”

(Kenya Environmental Sanitation and Hygiene Policy 2016–2030).

#### 3.1.5. Environmental Cleaning

Basic environmental cleaning in a healthcare facility was defined as:

“*Definition: Proportion of health care facilities which have protocols for cleaning, and staff with cleaning responsibilities have all received training on cleaning procedures*”

(Core Questions for monitoring WaSH in healthcare facilities in the SDG).

“*Housekeeping refers to the general cleaning of hospitals and clinics, including the floors, walls, certain types of equipment, furniture, and other surfaces. Cleaning entails removing dust, soil, and contaminants on environmental surfaces. Cleaning helps eliminate microorganisms that could come in contact with patients, visitors, staff, and the community; and it ensures a clean and healthy hospital environment for patients and staff.*”

(National Infection and Prevention and Control Guidelines for Health Care Services, 2010)

Environmental cleaning is a major challenge due to financial constraints:

“*As a result, health facilities often lack funds for capital infrastructure investments and ongoing operation and maintenance as well as for overlooked functions such as cleaning and waste management*”

(WaSH in HCF, Practical Steps to Achieving Quality Care).

The constitution of Kenya indicted the right to a clean environment by all citizens but does not specifically address healthcare facilities.

“*Every person has the right to a clean and healthy environment, which includes the right—f(a) to have the environment protected for the benefit of present and future generations through legislative and other measures, particularly those contemplated in Article 69*”

(Kenya Constitution).

### 3.2. WaSH in Healthcare Facilities and Universal Health Coverage

The importance of WaSH in connection to achieving SDG3 was highlighted in some of the documents:

“*Noting that without sufficient and safe water, sanitation and hygiene services in health care facilities, countries will not achieve the targets set out in Sustainable Development Goal 3*”

(A72_R7 WaSH in Healthcare Facilities Resolutions). Specifically, the role of WaSH in healthcare facilities in achieving quality care as part of the implementing and achieving universal health coverage was mentioned.

“*In addition, WASH in HCF is important for meeting several targets under SDG 3 (health for all) and in particular target 3.8 on universal health coverage*”

(Core Questions for monitoring WaSH in healthcare facilities in the Sustainable Development Goals).

Universal health coverage was framed to include both financial and quality care.

“*Universal health coverage (UHC) means that all individuals and communities receive the health services they need without suffering financial hardship. It includes the full spectrum of essential, quality health services, from health promotion to prevention, treatment, rehabilitation, and palliative care*”

(WaSH in HCF, Practical Steps to Achieving Quality Care).

However, the national level documents did not mention universal health coverage in line with WaSH in healthcare facilities, but did associate UHC with quality care:

“*Other projects include digitization of records and health information system; accelerating the process of equipping of health facilities including infrastructure development; human resources for health development; and initiating mechanisms towards universal health coverage*”

(Kenya Health Policy 2014–2030).

“*The goal of devolution in health is to enhance equity in resource allocation and enhance access to essential services by accelerating Universal Health Coverage (UHC) and improving quality service delivery for all Kenyans, especially those who need it most*”

(Planning, Budgeting Performing, Review Process Guide for Health Sector).

The national monitoring tool focused on the registration process of citizens for the UHC and the frequency of visits by patients to a healthcare facility:

“*What mechanisms are in place to identify those registered for UHC*”

(Final UHC Level 2 and 3 Final Supervision Tool).

### 3.3. WaSH in Healthcare Facilities and Infection Control

Access and functionality of WaSH in healthcare facilities were associated with infection control in healthcare facilities and beyond:

“*Recalling WHA68.7 (2015) on the global action plan on antimicrobial resistance, which underscores the critical importance of safe water, sanitation and hygiene services in community and health care settings for better hygiene and infection prevention measures to limit the development and spread of antimicrobial-resistant infections and to limit the inappropriate use of antimicrobial medicines, ensuring good stewardship*”

(A72_R7 WaSH in Healthcare Facilities Resolutions).

Infection prevention and control in healthcare facilities was defined as:

“*Infection prevention and control (IPC) is broadly defined as the scientific approaches and practical solutions designed to prevent harm caused by infection to patients and health workers associated with delivery of health care*”

(WaSH in HCF, Practical Steps to Achieving Quality Care).

Kenya has a guide on healthcare infection prevention and prevention:

“*These guidelines are intended to provide administrators and HCWs with the necessary information and procedures to implement IPC core activities effectively within their work environment in order to protect themselves and others from the transmission of infections*”

(National infection Prevention and Control Guidelines for Health Care Services in Kenya, 2010).

Infection control in healthcare facilities was also associated with waste management:

“*Strengthening infection prevention and control systems including health care waste management in all health facilities*”

(Kenya Health Act.21 of 2017).

### 3.4. WaSH in Healthcare Facilities and Safety

WaSH, infection control and prevention were also associated with the safety of the public, patients, caregivers and healthcare workers:

“*Every patient and every family member and facility staff who cares for them deserves a clean and safe health care environment with high quality water, sanitation, and hygiene services*”

(WaSH in HCF, Practical Steps to Achieving Quality Care).

Aside focusing on the safety of all who visit health care facilities, some of the documents also highlighted the safety of healthcare workers:

“*Strategies to protect health workers include the following: Implementing standard precautions, Immunizing all health workers against HBV, especially those working in health care settings, Providing PPE, Managing exposures in a timely manner, Eliminating unnecessary sharps and injections Successful implementation of these strategies requires an effective quality improvement or infection prevention and control committee (IPCC) with support from the hospital management team*”

(National Infection Prevention and Control Guidelines for Health Care Services in Kenya).

Some national documents highlight the provision of safe healthcare facilities, but did not link safety to WaSH nor explain what a safe working environment entail:

“*the right to a safe working environment that minimizes the risk of disease transmission and injury or damage to the health care personnel or to their clients, families or property*”

(Kenya Health Act No.21 of 2017).

### 3.5. Civil Disruptions and Climate Change Impacts on WaSH in healthcare facilities

The functionality of WaSH in healthcare facilities is impacted by climate change or weather patterns or civil disruptions. In the context of the national documents, the increased burden on healthcare facilities was highlighted:

“*Political instability in the Eastern Africa region and the subsequent in-migration of refugees into Kenya has the result of increasing the demand for health services in the country and raising the risk of spreading communicable diseases*”

(Kenya Health Policy 2014–2030).

The need to appropriately site infrastructure was mentioned:

“*The site should have proper drainage, be located downhill from any wells, free of standing water, and not be in a flood-prone area. The site should not be located on land that will be used for agriculture or development*”

(National Infection Prevention and Control Guidelines for Health Care Services in Kenya).

The impact of climate change was highlighted, but framed as a question in the WaSHFIT tool:

“*Do seasonality and/or climate change affect WASH services and are there plans in place to cope with this*?”

(WaSHFIT, A practical guide for improving quality of care through WaSH in HCFs).

### 3.6. WaSH in Healthcare Facilities and Disaster Risk Reduction

Measures to reduce or eliminate the impact of climate change, civil disruptions and anthropogenic activities at the healthcare facility were mentioned:

“*Buildings are designed and activities are organized so as to minimize the spread of contamination by the movement of patients, staff and careers, equipment, supplies and contaminated items, including healthcare waste, and to facilitate hygiene*”

(Essential Environmental Health Standards in Healthcare).

“*Care must be taken, when siting latrines, to avoid contaminating groundwater and risk of flooding*”

(Essential Environmental Health Standards in Healthcare).

The national documents mention DDR in light of the general public not specific to the healthcare and WaSH facilities.

### 3.7. WaSH in HCF and Emergency Preparedness and Response

Healthcare services are needed in times of disasters or disease outbreaks. The importance of WaSH in healthcare facilities as part of emergency preparedness was highlighted:

“*WASH services strengthen the resilience of health care systems to prevent disease outbreaks, allow effective responses to emergencies (including natural disasters and outbreaks) and bring emergencies under control when they occur*”

(WaSHFIT, A practical guide for improving quality of care through WaSH in HCFs).

The national monitoring tool mentioned emergency preparedness in terms of referral systems, functional emergency teams and the presence of ambulances for patient transportation to referral hospitals:

“*Emergency preparedness and Timely Response in facility and referral. Has there been any referral in the last one month? Do you have a functional emergency response team?*”

(UHC Level 2 and 3 Final Supervision Tool).

At the county level, the hospital preparedness did not include WaSH:

*Hospital Preparedness. Infrastructure—Numbers of hospitals with Casualty Departments, ICU, Bed capacity, morgue facilities. Human resource—well trained cadres (Basic Life Support, Advanced Cardiac life Support.) Contingency/response plan updated. Disaster emergency kits, medicine stockpiles. Community support- alternative treatment centers*

(Health and Nutrition Sector Contingency Plan, 2019)

### 3.8. WaSH in Healthcare Facilities and Stakeholder Engagement

WaSH in healthcare facilities stakeholders emerged in six (6) documents. The implementation of WaSH in healthcare facilities is a multi-stakeholder activity. At the National level:

“*However, WASH is not the responsibility of the Ministry of Health alone. Ministries of Water and Sanitation are critical for improving municipal WASH supplies and providing technical expertise to health care facilities. Ministries of Finance can provide important budget allocations and financing mechanisms. Moreover, local governments have a responsibility to manage and fund WASH at the local level. Overall, coordination requires a high level of leadership beyond any one ministry to ensure a common, cohesive approach*”

(WaSH in HCF, Practical Steps to Achieving Quality Care).

Specifically, quality health care services should be monitored:

“*The district health management team (DHMT) is responsible for monitoring the facilities within the district for using and complying with IPC practices. The DHMT is also responsible for ensuring that adequate and appropriate resources are available to support IPC practices within these facilities*”

(National Infection Prevention and Control Guidelines for Health Care Services in Kenya).

Other aspects of stakeholder engagement are training, monitoring and evaluation were mentioned.

“*Prepare a budget that reflects aims and available resources, with potential to scale-up. The training budget should realistically consider all the costs, which include the actual training, but also the followup support that is required to assist facilities in ongoing challenges and improvements. In addition, it is useful to consider the funds for physical supplies as even providing some minor, immediate improvements (such as hand hygiene stations, low-cost water filtration or on-site chlorine generation) can help realize major improvements in reducing health risks and set the foundation for longer term improvements such as piped water*”

(WaSHFIT, A practical guide for improving quality of care through WaSH in HCFs).

## 4. Discussion

Guided by the political ecology of health theory this paper explored the framing of WaSH in healthcare facilities in relevant policies, guidelines, legislation, plans, monitoring and evaluation documents at the global and national context using Kenya as a case study. In these documents, WaSH in healthcare facilities was framed in relation to the importance of WaSH in a healthcare facility such as infection prevention and control, quality care and achieving universal health coverage. It was also framed in terms of infrastructure in healthcare facilities. From a political ecology of health perspective, the global agenda on WaSH in healthcare facilities influenced the growing concerns of WaSH in healthcare facilities at the national level in Kenya. From this study, the global agenda on achieving the sustainable development Goal 3 and Goal 6 influenced political, social, economic and cultural factors in the implementation and use of WaSH in healthcare facilities in Kenya. The global resolutions, guidelines and monitoring documents are guides for national level adaption. Similarly, with respect to the influence of global campaigns on national agenda, Asiki et al. [19] established that the Kenya national guidelines on cardiovascular diseases were guided by existing global initiatives and guidelines such as the Tobacco control act. Specifically, the global campaign on achieving universal health coverage led by the World Health Organization accelerated movements to implementing universal health coverage in Kenya as stated in the Kenya health policy (2013–2030). Kenya is currently piloting universal health coverage in four counties. The acronym WaSH means water, sanitation, hygiene, waste management and environmental cleaning [1]. From this research comprehensive mention of WaSH in healthcare facilities was dominant in global documents than national documents. Two national documents mentioned water, sanitation and hygiene in sentences excluding environmental cleaning and waste management. other national documents mentioned one of these components. First, this could be associated with the fact that the global documents addressed WaSH in health care facilities specifically. None of the national documents were published specifically for WaSH in healthcare facilities. Second, most of the national documents were published before the agenda for WaSH in healthcare facilities was initiated. In addition, the final monitoring tool for universal health coverage does not comprehensively measure access and functionality of water, sanitation, hygiene, waste management and environmental cleaning. It monitored aspects of water and hygiene. Waste management, sanitation and hygiene are in the same category. For instance, the presence of a functional incinerator, a well-protected ash pit, a well-protected placenta pit and having a set of three color-coded bins in all wards and clinical departments and used for segregating waste at the point of generation are in the same category. At the time of data collection, a universal health coverage policy or agenda was not instituted. However, it was evident from the final universal health coverage monitoring tool for the Kisumu County that efforts towards the implementation of universal health coverage were directed towards finance and registration of citizens than quality care. Indicators for WaSH in healthcare facilities were not adequately presented and this could have impacts on the planning and financing of quality care when the universal health coverage program is fully rolled out in the country. Similarly, Maccord et al. [42] highlighted the need for quality data collection on relevant WaSH in healthcare indicators to achieve environmental health policies in healthcare facilities in their research in Malawi. In addition, inadequate or inconsistent data will complicate the assessment of interventions towards implementing universal health coverage [43]. It was also evident that the previous healthcare facility monitoring tool, titled the Integrated Management Supportive Supervision tool measured more WaSH in healthcare indicators than the final universal healthcare monitoring tool measured. Although this tool did not comprehensively cover all the aspects of WaSH, it touched on all five components of WaSH. For instance, the tool monitored separated toilets for staff and patients.

WaSH in healthcare facilities cannot be achieved without the relevant key stakeholders at both the national and global levels. Ensuring access to WaSH in healthcare facilities is complex and requires the efforts of different institutions. Forming partnerships are very critical to achieving complex and connected challenges [44]. The global documents such as the WaSH resolutions document listed some key institutions, ministry of health, water, finance and energy in achieving WaSH in healthcare. Other relevant key stakeholders include communities where healthcare facilities are situated and nongovernmental organizations. WaSH in healthcare facilities was also framed in terms of stakeholder engagements such as trainings. Training on WaSH management or infection control, budgeting of funds for implementing WaSH in healthcare services and monitoring and evaluations are some of the key roles of government and nongovernmental organizations mentioned in both the global and national documents. For instance, inadequate data collection has been associated with lack of technical knowledge on policy documents or monitoring tools by government officials [42]. This barrier hinders advocating for the appropriate resources required for effectively implementing environmental health policies and plans by civil society groups and non-governmental organizations. Maina et al. [3] in their research on the role of WaSH in healthcare facilities in averting anti-microbial resistance in 14 county level hospitals reported inadequate resource allocation by the government as a key challenge to accessing WaSH in healthcare facilities. Similarly, Guo & Bartram [20] reported that about a fifth of facilities overall 14 countries they investigated as part of a study to explore predictors of water quality in rural healthcare facilities reported having an insufficient budget for supplies for water, sanitation and hygiene or infection control. Resources or funding is a major requirement to implementing WaSH in healthcare facilities [45]. Anderson et al. [46] in their paper expressed the need for WaSH in healthcare facility stakeholders to adequately monitor the quality, quantity, input and output of WaSH services in healthcare facilities to ensure effective costing when planning for water, sanitation, hygiene, waste management and environmental cleaning in a healthcare facility. It is also recommended that WaSH national documents in SSA should include relevant stakeholders such as the cleaners and maintenance officers since they directly deal with issues of WaSH in a healthcare facility [46].

The importance of WaSH in healthcare facilities cannot be underestimated in terms of infection control and prevention and safety of facility users and workers. Cleaning and disinfection of healthcare facilities prevent disease transfer and if not adequately handled weakens the healthcare system. Similar to the Ebola outbreak, the current COVID-19 outbreak has compromised the quality of care in many healthcare facilities and a growing number of healthcare workers have died even in global north countries. However, WaSH is not listed as a requirement for hospital preparedness in the 2019 County level health and nutrition contingency plan. The issue of WaSH and safety of patients, caregivers and workers were dominant in global documents than the national documents. The national infection prevention and control guidelines for health care services in Kenya clearly lays out the procedures, roles and responsibilities in infection prevention and control at the health care facility. Other documents mentioned the need for ensuring a safe working environment for healthcare workers, but do not clearly define what a safe environment means. However, the previous monitoring tool for healthcare facilities monitored the presence of personal protective equipment such as the single use of aprons, goggles, gloves, fire extinguishers and fire exit.

The safety and functionality of WaSH services in healthcare facilities were also framed in the context of natural disasters such as drought and floods. Only the health act mentioned issues of WaSH in healthcare facilities in association with impacts of climate change. WaSH infrastructure and climate change is also framed as a caution to ensure WaSH infrastructure are efficient and can withstand and recover from the shocks of climate variability impacts. For instance, engaging in waste burial or burning in a flood prone area facilitates surface and ground water contamination. Civil disruptions such as political instability burdens the functionality of healthcare facilities and WaSH infrastructure in two ways. The structures are often destroyed or the healthcare facilities are burdened with people seeking healthcare. However, these civil disruptions are not mentioned in the global documents in the context of WaSH in healthcare facilities. Kenya has recorded several civil disruptions. Of most significance is the post-election violence in 2017. Civil disruptions need to be considered in WaSH in healthcare facility planning, implementation and maintenance. This brings to question the framing of WaSH and disaster risk reduction in healthcare facilities. Disaster risk reduction was framed as a recommendation to healthcare managers.

The universal health coverage policy was not available at the time of this study, the authors only had access to the final universal health monitoring tool for level 2 and level 3 facilities. This is a limitation of this study since the authors could not comprehensively analyze the framing of quality care as part of the universal health coverage campaign in the country. However, access to the UHC final monitoring tool highlights the indicators of UHC being prioritized during the piloting phase. This phase is critical to the finalization of the UHC policy in the country.

From a policy perspective, there is a need for the development of a national level WaSH in healthcare facility guideline which addresses contextual factors of Kenya across all levels of the healthcare system. All relevant stakeholders should be engaged in the development of a comprehensive binding document on WaSH in healthcare facilities. This is necessary because research has closely associated the prevalence of disease and poor health management to the lapses in government policies in Ghana than other countries [47]. Second, the final monitoring tool for universal health coverage needs to be revised to comprehensively measure water, sanitation, hygiene, environmental cleaning and waste management indicators in healthcare facilities using the global tools as guides. It will ensure effective data collection, planning and implementation of WaSH in HCF. For example, it is evident that integrating WaSHFIT training and supervision enhance quality service provision in healthcare facilities [48]. Similarly, researchers have contextualized some monitoring tools in WaSH in HCF research. Maina et al. [40] adapted and contextualized the WaSHFIT tool and developed WaSHFAST for the assessment of WaSH indicator performance in facilities beyond primary healthcare level. The authors developed a total of 65 WaSH in healthcare indicators relevant to monitoring WaSH in hospitals in limited resource areas. In addition, there are existing monitoring tools which can be useful in monitoring WaSH in HCF indicators. Patel et al. [49] review on WaSH in healthcare monitoring tool developed from 1991 to July 2018 recommended the need for more comprehensive and concrete WaSH in health care monitoring tools. A recent assessment by the USAID and Maternal Child Survival Program on the Kenyan Health Management Information Systems (HMIS) indicated that half of hospitals surveyed used an electronic medical record that was not linked to the District Health Information Software (DHIS2) in 2016 [50]. The HMIS and the DHIS2 could be instrumental in monitoring required WaSH indicators and quality services should relevant WaSH indicators be included. From this review, the District Health Management Team (DHMT) is responsible for monitoring all activities in healthcare facilities. Access, functionality, safety and availability of water, sanitation, hygiene, environmental cleaning and waste management indicators should be reviewed by the DHMT. Effectively monitoring the indicators of WaSH in HCF will efficiently prepare facilities for disease outbreaks and disasters. In addition, it is evident that Kenya has policies, plans and guidelines which when enforced can address the issues of quality healthcare facilities. For instance, the need to include WaSH infrastructure in healthcare facilities was published in the National Infection Prevention and Control guidelines for healthcare services in Kenya in 2010. This is again emphasized in the Kenya Environmental and Sanitation Policy, published in 2016. It is evident more needs to be done to ensure policies are fully implemented (42). Commitment by all state officials, nongovernmental organizations and civil society groups are needed to achieve quality care in healthcare facilities. A review of reports on global meeting on WaSH in healthcare facilities: from resolution to revolution and the WaSH in health care facilities stakeholder commitments indicated varied levels of commitments. Several partners such as non-governmental organizations and private institutions have made commitments to support Kenya through global/national/local advocacy, technical support, implementation, research and learning [51]. However, Kenya government or country was not listed in the country level commitment section of the report published in 2019 [52]. Commitment and prioritization of WaSH in healthcare facilities by the country’s institutions and leaders will accelerate achieving quality healthcare. Issues of WaSH in healthcare facilities should gain equal prominence as issues of financing curative measures in healthcare facilities in the yet to be implemented UHC policy across the country by 2022.

## 5. Conclusions

In summary, accessing quality healthcare services is a challenge especially in marginalized areas. The lack of access to water, sanitation, hygiene, environmental cleaning and waste management in healthcare facilities affect the quality of care provided. From this research, relevant documents addressing issues of WaSH in healthcare facilities, quality health services and universal health coverage at the global and national levels framed WaSH in healthcare facilities in terms of its importance, like infection prevention and control and enhancing universal health coverage and types of infrastructure. Factors such as climate change and civil disruptions that affect the access and use of WaSH in healthcare facilities were also highlighted and framed as precautions to healthcare managers. However, the national document did comprehensively covered issues of water, sanitation, hygiene, waste management and environmental cleaning. In addition, the global guidelines at the national level are not comprehensively implemented which will lead to recurrent insufficient data on WaSH in healthcare planning. The influence from the global level on universal health coverage implementation at the local level is positive, but efforts at the national level were directed at the number of citizens registering and medication supply. Efforts should also be directed towards ensuring healthcare facilities have the appropriate infrastructure for infection control and safety of healthcare facility users. Ensuring good health through providing care as stated in SDG 3 cannot be achieved without efforts to achieve WaSH, SDG 6 at a healthcare facility.

## Figures and Tables

**Table 1:** List of documents included in the research.

Document Title	Author	Scale	Type	Year	No. of Pages
Water, sanitation and hygiene in health care facilities (WaSH in healthcare resolutions)	WHO/World Health Assembly	Global	World Health Assembly Resolution	2019	5 pages
Essential environmental health standards in health care	WHO	Global	Guideline	2008	59 pages
Water and Sanitation for Health Facility Improvement Tool (WASH FIT), a practical guide for improving quality of care through water, sanitation and hygiene in health care facilities	WHO	Global	Guideline	2017	92 pages
Water, Sanitation and hygiene in health care facilities, practical steps to achieve universal access to quality care	WHO/UNICEF	Global	Guideline	2019	70 pages
Core questions and indicators for monitoring WASH in health care facilities in the Sustainable Development Goals	WHO/UNICEF	Global	Monitoring tool	2018	28 pages
Laws of Kenya, The constitution of Kenya	National Council for Law Reporting with the Authority of the Attorney General	National		2010	194 pages
The Health Act No. 21 of 2017	Republic of Kenya	National	Act	2017	72 pages
Kenya health policy (2013–2030)	Ministry of Health, Republic of Kenya	National	Legislation	2013–2030	87 pages
Planning, Budgeting and Performance Review Process Guide for Health Sector (Simple Guide to MTEF for Health Sector)	Ministry of Health, Republic of Kenya	National	Guide	2019	41 pages
Public Health Act (Chapter 242)	National Council for Law Reporting with the Authority of the Attorney-General	National	Act	Revised Edition 2012	71 pages
Kenya Vision 2030 (The popular version)	Ministry of State for planning, Republic of Kenya	National	Strategic plan	2007	32 pages
Water Act, Chapter 372	Ministry of Water and Irrigation	National	Act	Revised Edition 2012 (2002)	245 pages
National Infection Prevention and Control Guidelines for Health Care Services in Kenya	Ministry of Public Health Ministry of Medical Services	National	Guideline	2010	210 pages
Kenya Environmental Sanitation and Hygiene policy	Ministry of Health, Republic of Kenya	National	Policy	2016–2030	
Building Code	The local government (adoptive by-laws) (building) order 1968	National	Legislation		138 pages
Health and Nutrition Sector Contingency Plan	Ministry of Health	County	Plan	2019	38 pages
Universal Health Coverage Level 2 & 3 Final Supervision tool	Ministry of Health	County	Monitoring tool	2019	13 pages

**Table 2:** Coding framework.

Input		Activities	Output	Impact
Political commitment to WaSH in HCF	Political will Types of regulatory documents implemented	Planning and decision making to implement WaSH in HCFs WaSH training (infection control, WaSH infrastructure use and management) Healthcare facility assessment Identification of hazards, risk level and action needed Incremental improved plan developed Monitoring and evaluation	WaSH Infrastructure. (access, quantity, quality, safety, functionality, usable, cleanliness) Improved, basic, limited and no access of: Water Sanitation Hygiene Waste management Environmental cleaning Healthcare facility type: Level 1,2,3	Health improvements Quality of care (Universal health coverage) Community improvement Emergency Preparedness and resilience improvement Natural disruptions Civil disruptions
Financial Commitment to WaSH in HCF	Financial responsibility Categories of items to purchase Financial allocation to upgrade/improve WaSH infrastructure			
Human Resource	WaSH personnel HCF staff training Leadership Community participation Health committee Stakeholder engagement			

**Table 3:** Filled coding schedule for global-level documents.

Code/Document	Description of Code	WaSH in Healthcare Resolutions	Essential Environmental Health Standards in Healthcare	WaSHFIT, a Practical Guide for Improving Quality of Care Through Wash in Healthcare Facilities	WaSH in Healthcare Facilities, Practical Steps to Achieve Universal Access to Quality Care	Core Questions and Indicators for Monitoring Wash in Healthcare Facilities in the Sustaibable Development Goal
Water	Availability of water/types of water sources in a healthcare facility	1	41	21	6	12
Sanitation	Presence/types of sanitation facilities in the healthcare facility		23	10	1	11
Hygiene	Presence/types of hygiene facilities in the healthcare facility	2	40	13	20	11
Waste management	Presence/types of waste management facilities in the healthcare facility		31	22	26	7
Environmental cleaning	State of cleanliness of the healthcare facility compound		9	7	6	11
Safe environment	General Safety	1	14	2	3	1
	Health workers Safety	2	1	1	2	
	Patient Safety	2	1	1	3	
WaSH	Water, sanitation, hygiene, waste management and environmental cleaning of a healthcare facility	20	19	37	142	20
Healthcare facilities	Healthcare settings, facility, hospital, etc.	1	47	25	40	13
Natural disruptions on WaSH in healthcare facilities	Floods, drought effects on WaSH in health facilities	2	1	3	1	
Civil disruptions impact on WASH in healthcare facilities	Conflicts on WaSH in healthcare facilities					
Disaster risk reduction in health care facilities	Measures in place towards building resilience	1	6	3	3	
WaSH and Healthcare Stakeholder Engagement	Planning, Budgeting and implementing WaSH in healthcare facilities of relevant stakeholders	5	29	57	39	6
Disease prevention and control in health care facilities	Disease prevention in healthcare facilities	1	26		1	
Infection control in healthcare facilities		15	34	13	19	8
Universal Health Coverage		4	11	1		2

**Table 4:** Filled coding schedule for national-level documents.

Code/Document.	Description of Code	Laws of Kenya, The Constitution of Kenya	The Health Act No.21 of 2017	Kenya Health Policy (2030)	Planning, Budgeting and Performance Review Process Guide for Health Sector (Simple Guide to MTEF for Health Sector)	Public Health Act (Chapter 242)	Kenya Vision 2030 (The Popular Version)	Water Act (Chapter 372)	Universal Health Coverage Level 2 and 3, Final Supervision Tool
Water	Availability of water/types of water sources in a healthcare facility	1						1	2
Sanitation	Presence/types of sanitation facilities in the healthcare facility		1						
Hygiene	Presence/types of hygiene facilities in the healthcare facility		2						1
Waste management	Presence/types of waste management facilities in the healthcare facility		2	2					4
Environmental cleaning	State of cleanliness of the healthcare facility compound	1							
Safe environment	General Safety			1					
	Health workers Safety		1	1					
	Patient Safety			2					
WaSH	Water, sanitation, hygiene, waste management and environmental cleaning of a healthcare facility								
Healthcare facilities	Healthcare settings, facility, hospital, etc.	3	29	15	15	6	1		3
Natural disruptions on WaSH in healthcare facilities	Floods, drought effects on WaSH in health facilities					1			
Civil disruptions impacts on WASH in healthcare facilities	Conflicts on WaSH in healthcare facilities			1					
Disaster risk reduction in health care facilities	Measures in place towards building resilience	2	1	3			1		
WaSH and Healthcare Stakeholder Engagement	Planning, Budgeting and implementing WaSH in healthcare facilities of relevant stakeholders			3					
Disease prevention and control in health care facilities	Disease prevention in healthcare facilities			5		3			
Infection control in healthcare facilities			1	1		9			
Universal Health Coverage			2	6	2		2		5

**Table 5:** Filled coding schedule for national-level documents (continued).

Code/Document	Description of Code	National Infection Prevention and Control Guidelines for Health, 2010 Care Services in Kenya	Kenya Environmental Sanitation and Hygiene Policy 2016–2030	Building Code	Health and Nutrition Contingency Plan, 2019
Water	Availability of water/types of water sources in a healthcare facility	9			
Sanitation	Presence/types of sanitation facilities in the healthcare facility	2		1	
Hygiene	Presence/types of hygiene facilities in the healthcare facility	24			
Waste management	Presence/types of waste management facilities in the healthcare facility	25	29		
Environmental cleaning	State of cleanliness of the healthcare facility compound	9			
Safe environment	General Safety	2	1		
	Health workers Safety	11	1		
	Patient Safety	3			
WaSH	Water, sanitation, hygiene, waste management and environmental cleaning of a healthcare facility	2	5		
Healthcare facilities	Healthcare settings, facility, hospital, etc.				
Natural disruptions on WaSH in healthcare facilities	Floods, drought effects on WaSH in health facilities				8
Civil disruptions impacts on WASH in healthcare facilities	Conflicts on WaSH in healthcare facilities		2		
Disaster risk reduction in health care facilities	Measures in place towards building resilience				
WaSH and Healthcare Stakeholder Engagement	Planning, Budgeting and implementing WaSH in healthcare facilities of relevant stakeholders	3	12		2
Disease prevention and control in health care facilities	Disease prevention in healthcare facilities	2			
Infection control in healthcare facilities		56	2		
Universal Health Coverage					
